# PATHFINDER-CHD: prospective registry on adults with congenital heart disease, abnormal ventricular function, and/or heart failure as a foundation for establishing rehabilitative, prehabilitative, preventive, and health-promoting measures: rationale, aims, design and methods

**DOI:** 10.1186/s12872-024-03833-y

**Published:** 2024-03-26

**Authors:** Sebastian Freilinger, Harald Kaemmerer, Robert D. Pittrow, Stefan Achenbach, Stefan Baldus, Oliver Dewald, Peter Ewert, Annika Freiberger, Matthias Gorenflo, Frank Harig, Christopher Hohmann, Stefan Holdenrieder, Jürgen Hörer, Michael Huntgeburth, Michael Hübler, Niko Kohls, Frank Klawonn, Rainer Kozlik-Feldmann, Renate Kaulitz, Dirk Loßnitzer, Friedrich Mellert, Nicole Nagdyman, Johannes Nordmeyer, Benjamin A. Pittrow, Leonard B. Pittrow, Carsten Rickers, Stefan Rosenkranz, Jörg Schelling, Christoph Sinning, Mathieu N. Suleiman, Yskert von Kodolitsch, Fabian von Scheidt, Ann-Sophie Kaemmerer-Suleiman

**Affiliations:** 1 International Center for Adults With Congenital Heart Disease, Clinic for Congenital Heart Disease and Pediatric Cardiology, German Heart Center Munich, Technical University Munich, München, Germany; 2 Department of Cardiac Surgery, University Hospital Erlangen, Friedrich-Alexander-University Erlangen-Nürnberg, Erlangen, Germany; 3 Department of Cardiology, Medizinische Klinik 2 – Kardiologie und Angiologie University Hospital Erlangen, Friedrich-Alexander-University Erlangen-Nürnberg, Erlangen, Germany; 4 Clinic III for Internal Medicine, Faculty of Medicine and University Hospital Cologne, University of Cologne, Cologne, Germany; 5 Department of Paediatric Cardiology and Congenital Heart Disease Center for Child and Adolescent Health, Medical Center—University of Heidelberg, Faculty of Medicine, University of Heidelberg, Heidelberg, Germany; 6 Institute of Laboratory Medicine, Deutsches Herzzentrum München, Technical University Munich, München, Germany; 7 Department for Congenital and Paediatric Surgery, German Heart Center Munich, Technical University Munich, München, Germany; 8 Division for Congenital and Pediatric Heart Surgery, University Hospital of Munich (LMU), Munich, Germany; 9 European Pediatric Heart Center, Munich, Germany; 10 University Heart & Vascular Center, Hamburg, Germany; 11 Faculty of Applied Natural Sciences and Health, Division of Health Promotion, University of Applied Sciences and Arts Coburg, Coburg, Germany; 12 Helmholtz Centre for Infection Research, Biostatistics Research Group, Brunswick, Germany; 13 Department of Pediatric Cardiology, University Children’s Hospital, University of Tübingen, Tübingen, Germany; 14 General Medical Practice Martinsried, Planegg, Germany

**Keywords:** Congenital heart defects, Adult congenital heart disease, ACHD, Heart failure, Prospective registry, Patient outcomes

## Abstract

**Background:**

Adults with congenital heart defects (ACHD) globally constitute a notably medically underserved patient population. Despite therapeutic advancements, these individuals often confront substantial physical and psychosocial residua or sequelae, requiring specialized, integrative cardiological care throughout their lifespan. Heart failure (HF) is a critical challenge in this population, markedly impacting morbidity and mortality.

**Aims:**

The primary aim of this study is to establish a comprehensive, prospective registry to enhance understanding and management of HF in ACHD. Named PATHFINDER-CHD, this registry aims to establish foundational data for treatment strategies as well as the development of rehabilitative, prehabilitative, preventive, and health-promoting interventions, ultimately aiming to mitigate the elevated morbidity and mortality rates associated with congenital heart defects (CHD).

**Methods:**

This multicenter survey will be conducted across various German university facilities with expertise in ACHD. Data collection will encompass real-world treatment scenarios and clinical trajectories in ACHD with manifest HF or at risk for its development, including those undergoing medical or interventional cardiac therapies, cardiac surgery, inclusive of pacemaker or ICD implantation, resynchronization therapy, assist devices, and those on solid organ transplantation.

**Design:**

The study adopts an observational, exploratory design, prospectively gathering data from participating centers, with a focus on patient management and outcomes. The study is non-confirmatory, aiming to accumulate a broad spectrum of data to inform future hypotheses and studies.

**Processes:**

Regular follow-ups will be conducted, systematically collecting data during routine clinical visits or hospital admissions, encompassing alterations in therapy or CHD-related complications, with visit schedules tailored to individual clinical needs.

**Assessments:**

Baseline assessments and regular follow-ups will entail comprehensive assessments of medical history, ongoing treatments, and outcomes, with a focus on HF symptoms, cardiac function, and overall health status.

**Discussion of the design:**

The design of the PATHFINDER-CHD Registry is tailored to capture a wide range of data, prioritizing real-world HF management in ACHD. Its prospective nature facilitates longitudinal data acquisition, pivotal for comprehending for disease progression and treatment impacts.

**Conclusion:**

The PATHFINDER-CHD Registry is poised to offer valuable insights into HF management in ACHD, bridging current knowledge gaps, enhancing patient care, and shaping future research endeavors in this domain.

## State of research and scientific background

Adults with congenital heart defects (ACHD) constitute a profoundly medically underserved patient population on a global scale [1–3]. Approximately 50 million adults worldwide live with CHD, with over 360,000 individuals in Germany alone, and this number projected to escalate in the coming decades [4, 5]. Within Germany and Europe, the number is further augmented by a significant, albeit indeterminate cohort of refugees, asylum seekers, or migrants, among whom the incidence and prevalence of CHD are even more elevated.

Despite therapeutic advances, ACHD are chronically ill, characterized by significant residua or sequelae, which manifest both physically and psychosocially. Nearly all necessitate specialized, integrated care throughout their lifespan, differing markedly from the management of acquired heart disease.

### Residua, sequelae and complications in ACHD

Typical residua, sequelae and complications stemming from the underlying heart disease and its treatment encompass heart failure (HF)/ ventricular dysfunction, cardiac arrhythmias, pulmonary vascular disease, aortopathies, or infective endocarditis [2, 6–9]. Therapeutic interventions often precipitate cardiac remodeling, scarring, or myocardial stiffening, with ramifications extending to all other organ systems. Additional acquired cardiac and non-cardiac comorbidities further aggravate the long-term course, frequently precipitating psychological morbidities such as post-traumatic stress disorder (PTSD), depression, and anxiety disorders [10–12].

### Heart failure in ACHD

Globally, heart failure (HF) affects approximately 64 million patients and presents a burgeoning public health concern due to its attendant morbidity and mortality. In Germany, HF is among the top three causes of mortality, with a 5-year survival rate of 50% and nearly 35,000 deaths annually. Each cardiac decompensation and hospitalization worsens the prognosis and increases the mortality risk.

In ACHD, HF from chronic pressure or volume overloads, intracardiac scars, valvular heart disease, arrhythmias, pulmonary hypertension, or ischemia due to congenital coronary anomalies is the main reason for increased morbidity and mortality. Variables such as the duration and severity of cyanosis, type of cardiac surgery, late effects of heart–lung machine operation, cardio protection during operative treatment, age at treatment, and time since the procedure additionally modulate risk. Approximately 25% of afflicted individuals succumb from HF, with selected CHD witnessing mortality rates soaring to 50% [13–15].

The risk profile for HF is particularly pronounced in cohorts with univentricular hearts (post-Fontan operation), with systemic right ventricle after atrial redirection in transposition of the great arteries, with severe pulmonary vascular disease (Eisenmenger syndrome), or with profound heart valve dysfunction subsequent to repair of tetralogy of Fallot [8, 16]. Notably, robust data pertaining to HF management in CHD remain scarce, as these entities, typified by their complexity, have hitherto been interrogated only within limited cohorts so far, and frequently constitute an exclusion criterion in heart failure studies [17, 18]. Management recommendations from the corpus of acquired heart disease, where a wealth of evidence-based therapies exists, are only indirectly admissible.

### Special considerations and long-term care challenges for HF in ACHD

For individuals exhibiting systolic ventricular dysfunction of a morphologically left systemic ventricle, therapeutic strategies typically involve blockers of the renin-angiotensin system (RAS) such as ACE inhibitors and angiotensin-receptor blockers (ARB, sartans), angiotensin receptor neprilysin inhibitors (ARNIs), beta blockers, mineralocorticoid receptor antagonists (Aldactone, Eplerenone), as well as diuretics (loop diuretics, thiazides, metolazone), and digitalis glycosides [18, 19]. Emerging therapeutic avenues such as empagliflozin, dapagliflozin, or vericiguat, are encumbered by a dearth of data.

For the treatment of heart failure with preserved systolic function (HFpEF), our understanding of therapeutic modalities in the realm of CHD remains rudimentary [20].

The data on the treatment of ventricular systolic dysfunction of a morphologically right systemic ventricle (e.g. after atrial redirection in transposition of the great arteries, in congenitally corrected transposition, and in univentricular hearts of the right ventricular type) is also scant. This also applies to individuals with univentricular hearts and surgically created passive pulmonary blood flow (Fontan circulation) [21].

In refractory heart failure scenarios, where conservative therapy has been exhausted, resynchronization therapy, mechanical assist devices, and heart or heart–lung transplantation are fundamentally available options [14, 22]. However, the efficacy of these treatments is currently ambiguous, and long-term results are also lacking [23–25]. Heart or heart–lung transplantation is limited by the often complex anatomy of CHD and the shortage of donors [26].

In addition to inadequate, evidence-based treatment recommendations, knowledge about preventive, prehabilitative, and health-promoting measures that could positively influence the course of the disease, is virtually non-existent.

Current guidelines addressing cardiac rehabilitation in ACHD offer scant directives. Only small studies confirm the feasibility of such interventions and provide only cursory recommendations [27]. As a consequence, today a paltry proportion of patients avail themselves of rehabilitative or prehabilitative measures.

There is a marked deficit in patient education regarding their condition and the relevance and necessity of health-relevant behaviors [3, 28]. There is a tremendous ignorance among patients regarding available entitlements, responsible cost bearers, clinic selection, appeals processes, and similar domains [28, 29]. As a result, the utilization of rehabilitative or prehabilitative services remains suboptimal among affected individuals.

### Objectives and goals

The primary objective of this research project is to ameliorate the inadequate data situation regarding the care of ACHD. This ambition will be achieved by the establishment of a prospective, epidemiological-clinical registry dedicated to the management of heart failure in ACHD and abnormal ventricular function and/or anatomy.

The project intends to analyze the collected data to develop concepts for the evidence-based optimization and safe implementation of heart failure therapy.

Additionally, this initiative will lay the foundation for the integration of preventive, rehabilitative, prehabilitative, and health-promoting initiatives, tailored to the unique needs of individuals with ACHD.

Through a nationwide registry, gathered real world data will answer open questions in ACHD care, particularly elucidating the treatment and counseling imperatives across various stages of the disease.

A comprehensive delineation of key focal points is encapsulated in Table 1.

**Table 1:** Objectives of the PATHFINDER Registry and future perspectives

**Specific objectives**	
Drug therapy	Monitoring and documenting the therapeutic regimen, encompassing ACE inhibitors, AT blockers, ARNIs, beta-blockers, MRAs, diuretics, SGLT2 inhibitors, soluble guanylate cyclase stimulators (e.g., Vericiguat), digitalis glycosides, and pulmonary-vasoactive agents
Personalized precision medicine	Developing algorithms for personalized precision medicine through the utilization of pattern recognition methodologies
Medical devices	Monitoring and documenting treatment outcomes following implantation of pacemaker systems, cardiac resynchronization therapy, post-implantable cardioverter-defibrillator (ICD) interventions, and cardiac contractility modulation (CCM) devices
Interventions	Documenting post-procedural outcomes subsequent to interventional or surgical interventions
Assist devices	Monitoring and documenting patient progress with assistive devices
Transplantation	Documenting clinical course of patients awaiting transplantation, and after transplantation
Clinical warning signs	Evaluating safety profiles of therapeutic interventions and identifying risk patterns, including early indicators
**Future perspectives**	
Prevention	Development of preventive measures to avoid clinical deterioration and heart failure
Prehabilitation	Establishing appropriate prehabilitation programs within existing rehabilitation facilities to prepare patients for more complex cardiac operations or interventions with health-promoting measures
Rehabilitation	Developing and expanding appropriate rehabilitation measures within experienced facilities to optimize functioning and reduce disability in interaction with their environment
Disease programs	Integrating ACHD management into targeted preventive health programs

Further Goals Include:

1. Enhancing the health status of ACHD: The primary aim of the registry is to optimize current HF therapy, thereby fostering an amelioration in the well-being and health status of ACHD in Germany and potentially extending its impact beyond national borders.
2. A further goal is the establishment of an interdisciplinary platform for information exchange and dialogue. This collaborative forum would allow general practitioners and specialists from diverse clinical settings to exchange ideas on patient management strategies, treatment modalities, and on preventive measures against cardiovascular complications in ACHD. This collaborative effort aims to devise pragmatic approaches encompassing nutrition, exercise regimens, health promotion strategies, and psychoeducational interventions to optimize patient care. Addressing the deficiency in transition medicine services often associated with loss to follow-up and delayed presentations of ACHD patients with advanced complications is imperative.
3. Implementation of targeted training initiatives will increase awareness regarding the necessity for lifelong follow-up under the guidance of experienced ACHD specialists. This initiative seeks to instill routine provision of appropriate follow-up among healthcare practitioners managing patients with CHD.
4. Modelling the socioeconomic impact of health and health insurance, old-age pension insurance and early retirement in ACHD: Given the potential escalation in insurance costs and premature retirement associated with ACHD, modeling the impact of regular follow-up and preventive measures assumes paramount importance. The absence of comprehensive data on the economic burden of CHD underscores the necessity for modeling exercises to elucidate pathways for reducing disease-related costs, postponing retirement age through preventive interventions and regular follow-ups, and assessing the potential enhancement in both financial expenditures and quality of life through strategic interventions.
5. Transferring knowledge to rare disease communities: The insights gained from ACHD research holds promise for informing strategies applicable to other rare disease populations.
6. Paving the way for evidence-based clinical practice and foundation for future randomized controlled trials: This prospective registry serves as a foundational resource for future randomized controlled trials, facilitating evidence-based clinical decision-making and the formulation of corresponding clinical guidelines.

## Methods

### Ethical considerations and data protection measures

This registry operates in alignment with universally accepted ethical standards, encompassing the Declaration of Helsinki, as well as relevant local regulations and national laws.

Before initiating data collection as per the protocol, written informed consent is obtained from the patient, or their legally authorized representative, using the approved Informed Consent Form (ICF).

Comprehensive information regarding the registry, including its voluntary nature, will be clearly communicated to the patient through a direct conversation. Sufficient time will be provided for the patient to deliberate their participation in the registry. The signed ICF will be stored in the registry's records. The patient will receive a copy of the ICF, duly signed and dated. The patient, or their guardian, reserves the right to withdraw from the registry at any point. Those who choose to withdraw will not be substituted.

### Registration

The registry has been included in the German Clinical Trials Registry, DRKS (Ref. Nr.: DRKS00030508). An English version of the dataset has been submitted to the WHO Study Registry.

### Confidentiality and safeguarding data

Adherence to data protection regulations, including compliance with the General Data Protection Regulation (GDPR), is a priority. Neither patient initials nor exact birth dates will be entered into the database. Patient information is gathered using pseudonyms. When the baseline assessment is logged (at the inclusion visit), the Electronic Data Capture (EDC) system automatically generates a distinct and sequential Subject Identification Code. All registry-related documents, such as printed electronic case report forms and informed consent forms, are marked with that code. The investigator will keep a record for identifying patient records in response to inquiries. Data is exclusively transferred in an encrypted format.

### Design

PATHFINDER-CHD is a multicenter, prospective observational study. The registry allows for structured, non-interventional collection of data. There is no specified end date or minimum duration for the registry.

### Setting

The participating centers are among the largest specialist clinics in Germany for the care of ACHD. Physicians participating in the study retain full autonomy in diagnosing and treating their patients. Any examinations conducted are at the physician's discretion and based on their clinical practice.

### Patients

The designated population for the registry includes ACHD. There is no formalized process for screening potential patients. It is encouraged that physicians evaluate every patient with ACHD, to determine their suitability for inclusion in the registry.

Patients qualify for inclusion in the registry documentation if they:

- have any form CHD
- are aged 18 years or older
- have given informed consent (can also be provided by guardians)
- can be documented over a long-term follow-up period

Patients are not eligible if they participate in an interventional study, as this would interfere with real-world evidence generation.

Patients retain the flexibility to switch their medications and treatments as needed, even multiple times, throughout the documentation period.

### Data collection and quality control

The operation of the Electronic Data Capture (EDC) system, including the website and database, is managed by an institution with profound experience with registries. Data are entered by sites through a Hypertext Preprocessor (PHP)-based user interface into a Structured Query Language (MySQL) database. The raw data collected from the ACHD centers are securely stored in their original form in a central database. The data are backed up daily.

The raw data preparation and transfer to statistical analysis programs follow documented standards. These standards, as well as the data security and backup concept, are documented in the Standard Operating Procedures (SOPs) of the evaluating institute. During data collection, the documentation forms are transmitted to a central database by the study centers via direct electronic data capture over the Internet.

### Assessments

The registry is structured to include a fundamental set of variables (mandatory data) crucial for all patients enrolled. Additionally, there's a secondary set of variables (optional data) that may be solicited from participating facilities, though their provision is voluntary. The system is tailored to support sub-studies, which involve the incorporation of additional variables, at specific centers. This facilitates research collaboration among different institutions.

Baseline assessments and regular follow-ups are conducted whenever a patient visits the facility, undergoes therapy changes, or experiences events or complications related to their CHD. A list of all collected variables can be found in Table 2.

**Table 2:** List of variables collected in PATHFINDER-CHD

	**Inclusion**	**Follow-up visits**
**Parameter**		
Demographic data	X	
Type of congenital heart defect /anomaly Congenital anomalies of the left heart Congenital anomalies of the right heart Septal and vascular defects Complex cardiovascular defects Coronary artery anomalies Connective tissue disorders Congenital metabolic disorders with cardiovascular involvement Other inherited disorders with cardiovascular involvement	X	
Comorbidities: Cardiac and Non-cardiac	X	X
Post operative / postinterventional status Palliative cardiac surgery Corrective/reparative cardiac surgery Cardiac catheter intervention Antiarrhythmic cardiac intervention Follow-up: Re- and further Operations/-Interventions	X	X
Chronic heart failure (CHF) Type Number of decompensations Clinical course Signs and symptoms of decompensation Functional class ACC/AHA stages	X	X
Electrocardiogram	X	X
Echocardiography Heart anatomy and function assessment Assessment of cardiac valves Assessment of great arteries	X	X
Magnetic Resonance Tomography Heart anatomy and function assessment Assessment of cardiac valves Assessment of great arteries	X	X
Laboratory Assessments	X	X
Cardiac catheterization data	X	X
Cardio-pulmonary exercise test data	X	X
6-min walk distance data	X	X
Cardiovascular medication at entry and discharge Heart failure-specific medication (1) Antiarrhythmic medication Anticoagulation Pulmonary vascular/hypertension (PH)-targeted medication (2) Additional medical treatment		
Accompanying measures (3)	X	X
Complications during treatment	X	X
Listing for transplantation	X	X
Information on current hospitalization	X	X

(1) Congestive Heart Failure (CHF) specific drug treatment includes beta blockers, ACE inhibitors, AT blockers (sartans), neprilysin-sacubitril, aliskiren, diuretics, aldosterone antagonists, sodium-glucose cotransporter 2 (SGLT2) inhibitors, soluble guanylyl cyclase (sGC) stimulators, digitalis glycosides, levosimendan, PDE III inhibitors, milrinone and calcium channel blockers

(2) PH-targeted drugs included PDE-5-inhibitors, endothelin receptor antagonists, sGC stimulators, prostacyclins, sotatercept (after approval), oxygen-insufflation (O_2)_ therapy

(3) Accompanying measures include: physical therapy, cardiac devices including resynchronization therapy or ICD, lifestyle modification

### Data analysis and compliance

The analysis will mainly employ standard methods of descriptive and inferential statistics as well as machine learning techniques. Results based on samples will be reported with 95% confidence intervals. Additionally, time-to-event and predictor analyses are planned. The study is in accordance with the current version of the Declaration of Helsinki. All steps of the analyses will be conducted in compliance with Good Practice Secondary Data Analysis (GPS) [30].

The analysis of the registry data will be conducted globally across the entire registry and, if requested, can also be specific to individual institutions. The analysis includes age- and gender-specific evaluations. Standard methods of descriptive and inferential statistics will be employed. The results will be described using descriptive, exploratory and inferential statistical methods. These include the calculation of statistical epidemiological measures, determination of confidence intervals, and more. Data visualization will be achieved through histograms, scatter plots, cross-tabulations, and dimension reduction methods like principal component analysis, t-distributed stochastic neighbor embedding (t-SNE) or uniform manifold approximation and projection (UMAP) depending on gender.

A wide range of statistical and machine learning methods will be used for data analysis. This includes t-tests, analyses of variance (ANOVA), correlation analyses, regression analyses, latent variable/latent class models, latent growth models and time-to-event analyses. These methods will enable a comprehensive and detailed understanding of the data, facilitating the identification of patterns, relationships, and trends within the ACHD population. This approach ensures a thorough exploration of the data, allowing for robust conclusions and insights to be drawn from the registry.

## Results

At the present time, there are no results available.

## Discussion

The PATHFINDER-CHD registry will compile data from a minimum of 1.500 ACHD.

A key expected outcome is the improved early detection of complications through targeted risk monitoring, including follow-up care, based on specific criteria. This proactive approach promises to mitigate severe health consequences through earlier interventions. The data collected will also facilitate the development of more effective treatment and follow-up strategies, expected to enhance the prognosis for ACHD, thereby enhancing their quality of life and reducing mortality risks.

The research emphasizes the creation of preventive, prehabilitative, and rehabilitative measures grounded in the latest findings. These measures are expected to be directly implementable in clinical settings, offering a holistic and effective approach to patient care. The interdisciplinary care approach, informed by the registry data, is expected to significantly enhance patient safety by addressing the complex needs of ACHD in a tailored manner.

An integral part of the project is the establishment of interdisciplinary forums for continuous care, involving general practitioners and specialists. These forums will facilitate the exchange of knowledge and development of best practices for lifelong care of ACHD. By improving health outcomes, the project could delay or prevent the need for early retirement among ACHD, significantly impacting the economic burden on health care and pension systems.

Additionally, one of the foundational goals is to reduce disease-related costs for both statutory health insurance and pension insurance systems. Effective care strategies and early interventions can lead to substantial cost savings. Importantly, the insights gained from this patient cohort are likely to be applicable to other patient groups with rare diseases, extending the impact of this research beyond ACHD and paving the way for improved care strategies across a spectrum of less common conditions.

In conclusion, this project has the potential to improve substantially to the care of ACHD, offering not only improved clinical outcomes but also significant societal and economic benefits. The successful integration of these findings into clinical practice will be a crucial step in realizing these extensive benefits.

## Data Availability

Data sharing is not applicable to this article as datasets are being generated but have not been analyzed during the current study.
